# Perspectives From the 85+ Lifestyle Leaders on Social Isolation and Multigenerational Relationships

**DOI:** 10.1093/geroni/igab046.1069

**Published:** 2021-12-17

**Authors:** Taylor Patskanick

**Affiliations:** MIT, Somerville, Massachusetts, United States

## Abstract

The oldest of older adults are especially impacted by many of the measures recommended to slow the spread of COVID-19. This presentation explores changes in Lifestyle Leaders’ experiences with loneliness and their beliefs about the impact of COVID-19 on multigenerational relationships and intergenerational programming. For example, 55.6% strongly agreed or agreed with the statement, “The pandemic will have been more socially impactful on younger generations than older generations.” Lifestyle Leaders remain interested (68%) in virtual or socially distanced intergenerational programming. Particular activities of interest included technology tutoring, pen pals, and outdoor or virtual socializing. Additionally, this presentation will highlight how the Lifestyle Leaders have been impacted by a loss in weak ties and the extent to which the pandemic has prompted them to take on new roles in their families, including “accepting” paid and unpaid caregiving and experiences living with children and grandchildren during the pandemic.

